# Current landscape of vector safety and genotoxicity after hematopoietic stem or immune cell gene therapy

**DOI:** 10.1038/s41375-025-02585-8

**Published:** 2025-04-08

**Authors:** Giorgio Ottaviano, Waseem Qasim

**Affiliations:** 1 Pediatrics, Fondazione IRCCS San Gerardo dei Tintori, Monza, Italy; 2 Molecular and Cellular Immunology, University College London, London, UK

**Keywords:** Haematopoietic stem cells, Cancer immunotherapy, Lymphoma

## Abstract

Malignant transformation of gene modified haematopoietic stem cells caused anxiety following adverse events in early clinical trials using gamma-retroviral vectors (γRV) to correct haematopoietic stem cells (HSC) in monogenic immune disorders. Adoption of HIV-derived lentiviral vectors (LV) with SIN (self-inactivating) configurations greatly reduced risks and subsequently hundreds of patients have been dosed with HSC gene therapy for blood, immune and metabolic conditions. Nevertheless, as experience builds, it’s now well recognised that vector integration can drive clonal expansions and these may carry long term safety risks. Documented cases of haematological malignancy after SIN-LV gene therapy have recently emerged, in particular where heterologous retroviral promoters were employed and there are concerns around certain insulator elements and other possible contributors to clonal expansions. Similarly, tens of thousands of subjects have now received engineered T cell products, and longstanding dogma that mature T cells cannot be transformed is being questioned, with reports of a small number of malignant transformation events and wider concerns around secondary malignancies in some groups of patients. We summarize current clinical information and revisit genotoxicity risks following ex-vivo gene modification of HSC and T cells.

## Introduction

Since early proof-of-principle clinical trials demonstrated that incorporating restorative DNA into the genome of human haematopoietic stem cells (HSC) can treat inborn errors of immunity [1], a small number of advanced therapy medicinal products (ATMP) have secured regulatory approval. Gamma-retroviral vectors (γRV) were initially deployed to integrate recombinant expression cassettes into HSC, but despite initial encouraging results in clinical applications [2, 3], reports of inadvertent malignant transformation mediated by vector integration and proto-oncogene transactivation effects were an early setback [4]. Next generation applications used self-inactivating (SIN) γRV or HIV-derived lentiviral vectors (LV) which exhibited safer profiles in preclinical studies [5]. SIN-LV are now being widely applied for stable gene-addition into human cells and have been widely used for adoptive immunotherapies where the incorporation of chimeric antigen receptors (CAR) or recombinant T cell receptors (rTCR) have allowed T cell immune reprogramming against blood cancers [6, 7]. Additional recombinant gene-transfer applications in HSC have included treatments for haemaglobinopathies [8], immunodeficiencies [9] and rare metabolic conditions such as metachromatic leukodystrophy (MLD) [10], Hurler syndrome [11] and adrenoleukodystrophy (ALD) [12]. Pre-clinical and early human applications had suggested very low genotoxicity risks for such applications, but as larger numbers of subjects are treated and data accumulates, risks of genotoxicity, and the causes of direct or indirect secondary malignancies are now being revisited.

## Genotoxicity uncovered during gene therapy trials using haematopoietic stem cells (HSCs)

### Evidence of gamma-retroviral vector mediated genotoxicity

Initial γRV vectors employed split packaging systems but retained intact viral promoter and enhancer regions within their long-terminal repeats (LTRs) to support constitutional gene expression. Inborn errors of immunity were amongst the first human applications of γRVs to generate HSCs gene therapy and proved to be successful in correcting immunological defects in the first treated patients [1, 2]. Nevertheless, major safety concerns soon became apparent with leukaemic transformation events that were quickly attributed to vector insertion sites and transactivation of protooncogenes. In two early trials for X-linked Severe Combined Immunodeficiency (X-SCID) in France and UK aberrant clonal expansion and transformation of genetically modified HSC was observed, leading to acute lymphoblastic leukaemia in at least six patients [13–15]. Mapping of integration sites identified predominant integrations close to transcription start sites (TSS) and promoter regions of proto-oncogenes such as LMO2, CCND2, & BMI1. Longitudinal analysis found an increased predominance of particular integration sites at the time of leukaemia onset. Expression analysis corroborated associations between vector insertion sites and leukaemogenesis, with aberrant patterns of expression of proto-oncogenes identified. Notably, LMO2 expression was reported in 5 out of 6 patients across both trials and recently one of these subjects was reported to have developed a second, long delayed, T-cell leukaemia complication related to clones with a distinct LMO2 insertion pattern [16]. Further γRV genotoxicity emerged from other first-in-human gene therapy trials for Chronic Granulomatous Disorder (CGD) and Wiskott-Aldrich Syndrome (WAS). In subjects treated with γRV gene therapy for CGD there was evidence of transgene silencing and loss of polyclonality within months of therapy with emergence of progenitor cell clones harbouring vector integrations within three genomic sites (MDS-EVI1, PRDM16, SETBP1), with clustering in proximity of TSS [17]. Eventually these patients developed myelodysplastic syndrome (MDS) with chromosome 7 monosomy, driven by transactivation and overexpression of MDS-EVI1 complex locus (MECOM), a well-known proto-oncogene involved in the genesis of myeloid leukaemia and chromosomal instability [18]. Similarly, HSC gene therapy for WAS, resulted in 7 of 10 patients developing transformation events leading to haematological malignancies, including T cell acute lymphoblastic leukaemia (T-ALL) and acute myeloid leukaemia (AML). Integration site analysis revealed predominant vector integration sites within LMO2 (six T-ALL), MDS1 (two AML), or MN1 (one AML) loci [19].

A recent systematic review and meta-analysis documented 21 genotoxicity events following treatment with γRV vector across seven clinical trials for primary immunodeficiency, the majority attributed to trans-activation of LMO2 (nine patients) or MDS-EVI1 complex (six patients) [20]. In addition, there is a report of delayed onset T-cell leukaemia in a child treated for adenosine deaminase severe combined immunodeficiency (ADA-SCID) using Strimvelis^®^, the first γRV-gene therapy licensed in Europe [21]. Here, whole genome sequencing and RNA sequencing of blasts revealed multiple somatic variants, chromosomal translocations and genomic mutations, partially resembling the abnormalities in other cases of post-gene therapy T-cell leukaemia [22]. Interestingly, a clone harbouring an activating vector insertion within the MECOM gene locus was also detected, having emerged during a course of chemotherapy, suggesting a potential “clonal advantage” was derived following vector integration. Until this case was reported, there had been speculation that compared to other immunodeficiencies, gene therapy for ADA-SCID may be safer, but longer term follow-up will provide a deeper insight into mechanisms and contributing factors for malignant transformation [23].

### Revised vector designs for improved safety

To date, two main features of γRV vectors have been of concern: (i) Transactivating effects of powerful retroviral enhancer elements embedded into the LTRs; (ii) Vector integration preferences for TSS, especially when in proximity of proto-oncogenes [24]. Self-inactivating vectors, with deleted U3 promoter regions and reliant on heterologous promoters and derived from LV were found in preclinical animal studies to have reduced transformation risk compared to γRV employing intact LTRs [25, 26]. Patterns of integration favoured active genes but were not skewed towards proto-oncogene regions, and high copy numbers and robust gene expression in haematopoietic stem cells was supported [27–29]. Various additional lentiviral design modifications have been investigated in the hope of improved safety, including incorporation of genetic insulators, both as barriers against repressive effects of neighbouring heterochromatin and to reduce trans-activation of nearby promoters by heterologous enhancers [30]. Post-transcriptional regulatory elements (PRE) derived from Woodchuck Hepatitis, included to increase transgene expression and stability and reduce transcriptional read-through, were modified and start-site disrupted to reduce risks [31, 32]. In an early clinical trial adopting SIN-LV for β-globin gene transfer into HSCs for gene therapy for ß-thalassemia, therapeutic benefit was achieved enabling transfusion independence [33]. Integration site monitoring observed a progressive predominance of vector insertion into the HMGA2 gene in myeloid cells, that led to the upregulation of HMGA2 expression in erythroblasts and clonal dominance. Analysis found aberrant insulator sequences and detected truncated HMGA2 transcripts created by alternative splicing that were insensitive to degradation. In this case, clonal expansion driven by gene addition probably contributed to the therapeutic goal, and subsequently clonal dominance retreated with no eventual severe adverse events reported. This event highlighted that while SIN-LV were less likely to mediate genotoxicity through LTR activity, they still harbour risks, including through the formation of chimeric or aberrant splicing and premature transcript termination [29]. Hundreds of stem cells therapies have since been delivered after SIN-LV modification in both Europe and US across multiple studies. Two cases of leukaemia were reported after LV-gene therapy for sickle cell disease in the HGB-206 Phase I/II clinical trial [34–36]. One patient had developed MDS with chromosome 7 monosomy three years after gene therapy, but integration site analysis did not reveal any absolute or relative clonal predominance over time. With no vector copies detected in leukemic blasts, insertional oncogenesis was considered unlikely, and other factors such as busulfan conditioning chemotherapy and stressed haematopoiesis in a sickle cell background may have been contributory [34]. A second patient diagnosed with AML was found to harbour vector integrants, with skewed insertion into the VAMP4 gene locus (not a known proto-oncogene) as well as mutations in RUNX1 and PTPN11, two genes associated with AML/ALL [35]. At the time investigators thought it unlikely that transformation was directly related to insertional mutagenesis, but was rather attributed to an increased underlying risk of malignancy of SCD patients after chemotherapy [37]. A subsequent cohort of patients receiving reduced busulfan doses reported that two subjects developed anaemia associated with trisomy of chromosome 8 [38], and another clinical trial using a SIN-LV containing a short-hairpin RNA targeting BCL11a to disrupt repression of foetal γ-globin [39], found genetic aberrations in HSC from six SCD patients, albeit without evidence of clonal dominance [40]. A study of SIN-LV gene therapy for SCID-X1 using vectors with insulator elements also reported clonal dominance during reconstitution, although without overt transformation [41, 42].

More recently, adverse events have been reported after HSC gene therapy for X-ALD using elivaldogene autotemcel (eli-cel) [43], where 7 patients were subsequently diagnosed with myeloid malignancies: MDS with uni-lineage dysplasia in 2 patients at 14 and 26 months; MDS with excess blasts in 3 patients at 28, 42, and 92 months; MDS in 1 patient at 36 months; and AML in 1 patient at 57 months. Investigations had revealed frequent lentiviral vector integration into proto-oncogenes at sites such as MECOM (resulting in increased expression of EVI1) and presence of somatic mutations (KRAS, NRAS, WT1, CDKN2A or CDKN2B, or RUNX1) in 6 out of 7 patients with leukaemia, while one patient had chromosome 7 monosomy. Notably, although a SIN-LV configuration was used, the internal promoter was a hybrid retroviral construction, MND (MPSV-LTR, NCR deleted, dl587 PBS), comprising a U3 region from the LTR of Moloney Leukaemia retrovirus and an enhancer derived Myeloproliferative Sarcoma retrovirus. This strong promoter had supported high levels of enzyme replacement but recapitulated previously reported transactivation risks of γRV promoter/enhancer elements [44–46].

## Ex vivo gene modification of immune cells and risk of transformation

Engineering of T cells, NK and other immune cells with chimeric antigen receptor (CAR) or recombinant T cell receptor (rTCR) to redirect immunity against tumour has been widely investigated for adoptive immunotherapy for over two decades [47, 48]. A number of authorised CAR-T cell products are available; Kymriah®, Carvykti®, Breyanzi®, Abecma® have adopted a third-generation SIN-LV, while Tecartus®, Yescarta® have used an LTR intact γRV for CAR gene transfer. As multiple groups around the world are exploring the development of new CAR-T cell therapy for B cell and non-B cell malignancies, data on the safety of genetic engineering of immune cells is accumulating.

### Considerations for vector risk in HSC and immune cells

Compared to HSC, the risk of malignant transformation during gene therapy using T cells was considered to be extremely low for several reasons: (i) Greatly reduced plasticity of differentiated immune cells compared to HSC; (ii) Transduction using γRV or LV required low multiplicity of infections (MOI) compared to much higher MOIs required for HSC; (iii) therapeutic strategies using T cells were considered to be less dependent on long-term persistence for their therapeutic benefit. Pre-clinical data suggested that T cells were highly resistant to transformation after insertional mutagenesis compared to HSC [49], and immortalisation of T cells was only reliably achieved following transduction with certain LTR intact vectors, for example when encoding LMO2, and if associated insertional activation of IL2RA and IL15RA for sustained immortalisation [50]. A ‘multiple hit’ model was exemplified in murine T cells when γRV insertional mutagenesis was detected in cells with additional aberrant signalling due to over activation of Janus Kinase-1 [51].

### Evidence of vector-driven T cells clonality

Tens of thousands of patients have now been dosed with engineered T cells, and long-term follow-up data have so far confirmed that transformation risk as a result of vector mediated genotoxicity appears low [52]. Nevertheless, there have been a number of reports of potential safety issues, warranting careful consideration. Initially, monitoring of patients treated with γRV modified T cells found variable levels of skewed integration patterns with preferential insertion within or near promoters of transcribed genes as anticipated, but no evidence of unstable gene expression or new-onset clonal dominance for up to 9 years after treatment [53]. Similarly, safety monitoring over 10 years in HIV patients treated with engineered CD4+ cells had found no evidence of T cell clonality or integration sites in genes involved in cell growth [54]. More recently, evidence of clonal expansion and dominance of SIN-LV-modified CAR-T cells have emerged in clinical trials in leukaemia patients. For example, one patient with chronic lymphoid leukaemia (CLL) who had received two infusions of CTL019 CAR-T cells experienced a delayed CAR T cell expansion that was found to be clonal [55]. Deep sequencing revealed skewed predominance of a single T cell clone, representing >90% of CAR T cells, which was not detectable in the pre-infusion product and was subsequently found to harbour a SIN-LV insertion into the methylcytosine dioxygenase TET2 gene site, a known tumour suppressor gene. Vector integration disrupted TET2 gene expression by alternative mRNA splicing, and this arose in the context of a pre-existing hypomorphic mutation carried by the other TET2 allele. Phenotypically the clone displayed central memory subset features, consistent with an altered epigenetic profile, and was ultimately associated with beneficial anti-leukaemic effects. Subsequently, TET2 has been noted to be a common lentiviral integration accounting for up to 1% of transduced cells and in pre-clinical models of TET2-targeted CAR-T cells, disruption of TET2 skewed differentiation of CAR-T cells towards an exhausted phenotype with preserved central memory features [56].

Corroborating the hypothesis that integration patterns in T cells might be implicated in clonal expansion, and ultimately associated with clinical responses, an extended integration analysis in 39 patients treated with anti-CD19 CAR-T cell therapy for B-ALL and CLL, suggested that recurrent vector integrations in genes associated with cell-signalling and chromatin modification were major drivers of clonal T cell proliferation [57]. Clonal expansion of CAR + T cells was also observed in another clinical trial, where lentiviral CAR T cells against CD22 T cells were investigated to treat B-ALL and a CAR-T surge was noted more than a month after an initial transitory CAR-T cell peak, and this coincided with disease clearance [58]. Integration site analysis revealed the emergence of a dominant clone that was not detected in the pre-infusion product and tracked to insertion into an intron of the CBL gene site, a regulator of TCR signalling and T cell activation. Longitudinal analysis showed that this clone was detectable in a low percentage from day+7 after infusion, but became predominant only during the second CAR-T expansion peak.

### T cell lymphomas in patients receiving CAR-T therapy

Secondary malignancies in patients treated with CAR-T cells have been estimated to occur in <5% of patients. In long-term follow-up reports these included MDS/AML and solid tumours [52, 59, 60]. A meta-analysis of cases of secondary malignancies in patients treated with CAR-T cells for lymphoma and multiple myeloma highlighted comparable incidences of secondary neoplasms in patients receiving CAR-T cell therapy or after conventional therapy. The incidence of T-cell lymphoma was reported as low as 1.5%, over 5517 patients receiving CAR-T cell identified [61]. A causative link with the process of T cell engineering in the development of these events was not established, and concurrent risk factors such as chemotherapy, radiotherapy or transplant may explain these events. The overt risk of transformation from γRV and LV-CAR products was highlighted to practitioners in November 2023, when the Food and Drug Administration (FDA) communicated concerns around 22 cases of T-cell lymphoma that had arisen in patients treated with commercially available CAR-T cell products [62, 63]. Event monitoring for commercial CAR-T cell therapies had screened 12,394 reports and identified 536 primary malignancies (4.3%). The most frequently reported neoplasms were MDS (38.8%), myeloid leukaemia (19.8%), while T-cell lymphomas were 3.2% of second primary tumours, with an overall prevalence of 0.1% of all CAR-T reports [64]. CAR transgene was reported to have been detected in the malignant clones in 3 out of 22 cases. Around the same time details of T-cell lymphoma in a 51-year old male treated for refractory/relapsed multiple myeloma, with Ciltacabtagene Autoleucel (Cilta-cel, LV anti-BCMA CAR T cells, Carvykti ®) [65], a licenced anti-BCMA (B-cell Maturation Antigen) SIN-LV CAR-T cell therapy were reported with evidence of T-cell clonality 5 months after a second wave of CAR-T expansion with lymphadenopathy and new onset skin lesions suggestive of T-cell infiltration. T-cell lymphoma was confirmed on histology, with high levels of CAR-transgene integration in malignant cells. Integration site analysis revealed insertion into the 3′UTR (untranscribed region) of the *PBX2 homeobox gene*, a transcription factor with known involvement in tumorigenesis, albeit without elucidation of direct causality. Analysis of the TCR signatures confirmed the underlying clone was present in the pre-infusion drug product and whole genome sequencing revealed additional genomic aberrations in TET2, PTPRB and NFKB2 and germline testing revealed the patient carried a heterozygous variant of the signalling gene, JAK3. Authors concluded that the pathogenesis was probably attributable to multiple genetic aberrations, possibly in a pre-existing T cell clone, in association with vector integration effects [66]. Further anecdotal cases of T-cell lymphomas are accumulating, but investigations to understand the link between T-cell manipulation and secondary malignancy may not be comprehensive [67, 68]. Another case of T-cell lymphoma was described among more than 400 patients treated with anti-CD19 and anti-BCMA commercial CAR-T cell therapy at the University of Pennsylvania [69]. Three months after receiving anti-CD19 CAR T cell therapy for B-cell non-Hodgkin lymphoma (B-NHL), a biopsy of a thoracic lymph, performed as staging for lung cancer, showed clonal CD8 + T cell infiltrate, with very low levels of CAR transgene detected. Tracking of molecular signatures allowed identification pre-existing T cell clones both in pre-infusion blood samples and in lung tissue. In June 2024, two other cases of T-cell lymphoma after commercial CAR-T cell therapy were reported, and highlighted challenges attributing pathogenicity to T cell engineering or vector integration. A case of T-cell lymphoma was reported among 724 patients treated at Stanford Cancer Institute [70]. Here, a 59-year-old patient with DLBCL developed a lethal EBV-positive T-cell lymphoma 54 days after Axicabtagene Ciloleucel (Axi-cel, γ-RV anti-CD19 CAR-T cells, Yescarta®) therapy. Pancytopenia after CAR19 T-cell therapy was associated with nodal and marrow infiltrates of T-cell lymphoma, with rapid disease progression. In-depth profiling identified shared mutations in TET2 and DNMT3A (DNA methyltransferase 3 alpha) genes in both B and T cell tumours which suggested a common clonal origin, although molecular signatures later discerned two distinct malignancies. The CAR vector was not detected in the malignant T cells at the genomic or protein level. Another case of CAR-negative angio-immunoblastic T cell lymphoma was diagnosed 4 months after Axi-cel therapy for NHL, showing pathogenic mutations in TET2 and DNMT3A, similarly to the previous case [71]. Although details on the role of pre-existing mutations or differences between B and T cell malignant cells were not provided, the authors speculated that clonal haematopoiesis could have played a role in triggering emergence of these mutations and whether an inflammatory milieu following cytokine release syndrome accelerated or contributed to lymphomagenesis.

These two cases inform the need to not only investigate vector integrations in newly diagnosed malignancies, but to also consider deeper multi-parameter profiling of tumour populations to define underlying mechanisms.

Conversely, another report described an indolent CD4 + CAR + T cell lymphoma developing 5 months after treatment for multiple myeloma with cilta-cel. RNA sequencing detected the CAR transcript in cancer cells and integration site analysis revealed insertion into *SSU72* gene [72]. A further case of peripheral T cell lymphoma (PTCL) occurred one month after Tisagenleceleucel (Tisa-cel, LV anti-CD19 CAR T cells, Kymriah®) infusion in a patient with primary central nervous system lymphoma [73]. Immunohistochemistry revealed high expression of CAR in the tumour tissue. Whole genome sequencing (WGS) detected disruptive mutations of TET2 and DNMT3A genes which had been present since the collection of CD34+ cells for autologous stem cell transplantation around 9 months previously, and in the T cell apheresis collected for CAR-T manufacturing. A second allelic mutation in TET2 was detected at very low levels both in the T cell apheresis and manufactured CAR-T cells before infusion. Integration site analysis in the CAR + PTCL revealed enrichment in 3 sites (DPF2, RAB11FIP3 and NPLOC4), most likely related to multiple vector integrations in a single clone. However, these genes are not usually associated with leukaemogenesis and while the underlying mechanism of uncontrolled clonal expansion of CAR+ cells remains unclear, it might be hypothesised that PTCL emerged as a result of clonal haematopoiesis driven by pre-existing somatic mutations.

Vector integration was also observed in a duodenal lymphoma, two months after treatment for multiple myeloma with cilta-cel [74]. T cell receptor and vector integration site analysis revealed clonal dominance over time with dominant clones harbouring integrants in gene loci for TANGO2 and TP53. p53 expression was markedly reduced in T cells, and additional underlying findings included a pre-existing nonsense mutation in DNMT3A but also a nonsense mutation in SOCS1, a negative regulator of Janus kinase (JAK) & signal transducer and activator of transcription (STAT) signalling, known to have casual associations with B cell lymphoma genes.

Finally, Harrison et al. recently reported another case of CAR-T-associated lymphoma in a woman receiving cilta-cel for multiple myeloma, who developed a T cell lymphoma harbouring the CAR transgene 16 months after infusion, concomitant with a new clonal expansion of CAR + T cells in peripheral blood [66]. Integration site analysis revealed a predominant insertion in ARID1A, a chromatin-regulating gene, with tumour-suppression function and involved in altered T cell differentiation, although no clear evidence of dysregulated expression was noted and contribution to lymphomagenesis of vector integration remains undefined. Of interest, similar to another previously described case [65], (reported by the same group) this patient exhibited biallelic alterations of TET2 (pre-existing allelic variant and de novo deletion). This again supports the notion that pre-existing somatic TET2 mutations in normal T cells may contribute to clonal expansion in CAR T cells.

Given the concern around T cell lymphomas (details summarised in Table 1), recent studies have provided important denominator information. One retrospective analysis of more than 700 patients enroled in 38 T cell trials, reported only one case of T cell neoplasia and there was no confirmation of vector integration [75]. Tracking of large cohorts will be pivotal to fully understand risks and clinical implications of transformation events, underlying the need for comprehensive long-term monitoring and reporting.

**Table 1:** Summary of published cases of genotoxic events in patients receiving haematopoietic stem cells gene therapy and CAR-T cell therapy.

Haematopoietic Stem and Progenitor Cells					
Disease	Vector platform/Genome editing	Genotoxic event	Patients reported	Suspected pathogenic mechanism	Refs.
X-SCID	γ-RV	ALL	6	Insertional mutagenesis: LMO2, CCND2, BMI1	[13]
CGD	γ-RV	MDS	5	Insertional mutagenesis: MDS-EVI1, PRDM16, SETBP1	[17, 111, 112]
WAS	γ-RV	ALL/AML	9	Insertional mutagenesis: LMO2, MDS1, MN1	[19]
ADA-SCID	γ-RV	ALL	1	Insertional mutagenesis: MDS-EVI1 Somatic variants Chromosomal translocations	[21, 22]
X-ALD	LV	MDS	7	Insertional mutagenesis: MDS-EVI1 Somatic mutations: KRAS, NRAS, WT1, CDKN2A, CDKN2B, RUNX1	[43, 46]
SCD	LV	AML/MDS	2	Conditioning chemotherapy Insertional mutagenesis: VAMP4 Somatic mutations: RUNX1, PTPN11	[35]
	CRISPR-editing HDR	Bone marrow aplasia	1	Unknown	[108]

Somatic T cells					
Multiple Myeloma	LV BCMA-41BB-CD3z (Cilta-cel)	TCL	1	PBX2 vector integration. Somatic mutations: TET2, PTPRB, NFKB2; Germline mutations: JAK3	[65, 66]
	LV BCMA-41BB-CD3z (Cilta-cel)	TCL	1	SSU72 gene vector integration; Multiple genomic changes	[72]
	LV BCMA-41BB-CD3z (Cilta-cel)	TCL	1	TANGO & TP53 vector integration SOCS1 and DNMT3A mutations	[74]
	LV BCMA-41BB-CD3z (Cilta-cel)	TCL	1	ARID1A vector integration Pre-infusion TET2 somatic mutation	[66]
NHL	γ-RV CD19-CD28-CD3z (Axi-cel)	TCL	1	JAK3 variant in pre-infusion T cell clone	[69]
	LV CD19-41BB-CD3z (Tisa-cel)	TCL	1	Somatic TET2/DNMT3A mutations pre-infusion	[73]
	γ-RV CD19-CD28-CD3z (Axi-cel)	EBV^+^TCL	1	Pre-infusion TET2 mutated clone	[70]
	γ-RV CD19-CD28-CD3z (Axi-cel)	AITL	1	Somatic TET2/DNMT3A mutations	[71]
	*pb* Transposon CD19-41BB-CD3z	TCL	2	Abundant copy number variations in proto-oncogene and onco-suppressors; High VCN	[85]
	LV TALEN-editing CD19-41BB-CD3z	Bone marrow aplasia	1	V(D)J recombination	[100]

*ADA-SCID* adenosine-deaminase severe combined immunodeficiency, *AITL* angioimmunoblastic T-cell lymphoma, *AML* acute myeloid leukaemia, *ALL* acute lymphoblastic leukaemia, *CGD* chronic granulomatous disorder, *EBV* Epstein-Barr Virus, *HDR* homologous DNA repair, *LV* lentiviral vector, *MDS* myelodysplastic syndrome; *pb* piggyBac, *RV* retroviral vector, *SCD* sickle cell disease, *TCL* = T cell lymphoma, *WAS* Wiskott-Aldrich syndrome, *X-ALD* X-linked adrenoleukodystrophy, *X-SCID* X-linked severe combined immunodeficiency.

## Interplay of contributing risk factors

**Fig. 1:**
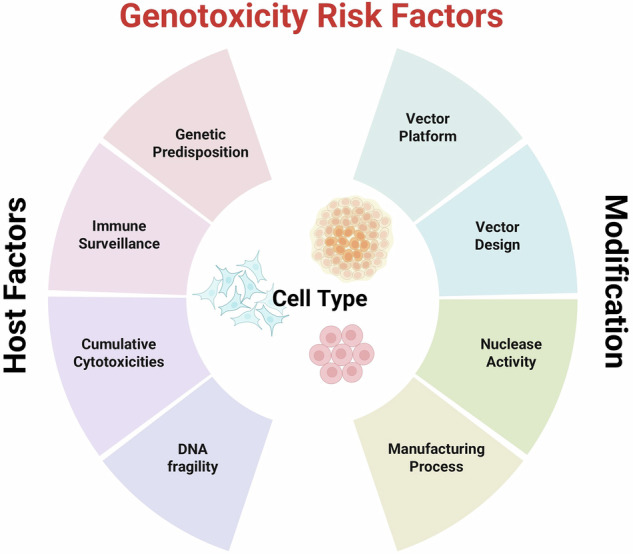
Risk factors for genotoxicity & transformation. This figure summarises potential risk factors for the development of malignancy after possible transformation events in engineered human cells, noting that underlying plasticity and stemness may be a critical factor, with more differentiated populations such as T cells less likely to transform than haematopoietic stem cells or pluripotent stem cells: (i) Host Factors: underlying genetic pre-dispositions may involve chromosomal changes or specific genetic mutations in pathways controlling cell signalling, proliferation, apoptosis or particular checkpoint pathways. Such changes may constitute an additional ‘hit’, operating in synergy with other events as they arise to drive clonal expansion; Defective immune surveillance due to underlying conditions or following immunosuppressive chemotherapy may result in a failure to recognise or effectively eliminate transformed populations; Cumulative toxicities may deliver ‘multiple hits’ following intensive chemotherapy, radiotherapy or after allogeneic stem cell transplantation; Underlying DNA fragility or DNA repair defects, are recognised as predisposing to cancer development, often involving multiple lineages beyond the haematopoietic system. (ii) Engineering Platforms: Integrating gamma retroviral and lentiviral vector platforms favour gene rich regions with predispositions towards transcription start sites and active genes respectively. Transactivation of proto-oncogenes, disruption of miRNA binding sites and other mechanisms may lead to insertional effects, and may be compounded by pre-existing mutations in certain circumstances. These risks may be influenced by the choice of endogenous promoter, with concerns around strong retroviral promoter and enhancer elements. There may also be risks related to high vector copy numbers or manufacturing processes with multiple rounds of gene transfer. The inclusion of nucleases for genome editing effects, including site-directed gene insertions, may be associated with predictable or unpredictable chromosomal changes. Alternative base editing or prime editing platforms may also mediate changes beyond those predicted at the DNA level.

In addition to vector design and integration effects other contributory factors may include the nature and function of a particular transgene, consequences of unregulated expression and in the case of chimeric receptors, the choice and configuration of activation domains. There is a possibility that impaired immune surveillance in patients with underlying immunodeficiency or impaired immunity after chemotherapy might be confounding factors (Fig. 1).

Previous lines of treatment and how they are tolerated in the context of underlying predispositions, such as DNA fragility or sensitivity to chemotherapy or radiation are also likely to be important contributing factors.

Somatic mutations in genes encoding for epigenetic modifiers (TET2, DNMT3A, ASXL1) have been identified as potential drivers of clonal haematopoiesis (CH) [76–79] and are associated with increased risks of blood cancers [80]. CH has been a common pre-infusion finding in patients receiving CAR-T therapy and is being investigated as a factor for secondary cancers after CAR-T therapy, with reported TET2 and DNMT3A mutations in recent descriptions of T cell malignancies [65, 71, 73, 74] Following infusion of BCMA-CAR-T cell (cilta-cel) a secondary expansion of clonal CAR-T cells, associated with fatal liver toxicity, has also been described. A somatic heterozygotic TET2 mutation found in the CAR T cell clone, was also present in the apheresis product [81], and lentiviral integration in ETS1 was apparently not associated with altered transcription. It has also been speculated that there are links between CH and post infusion cytokine release syndrome (CRS) [82] & it is plausible that a pro-inflammatory environment may be associated with transformation of pre-existing clones [71, 83].

## Alternative platforms and emerging genome editing tools: more possibilities bring greater responsibilities

### Non-viral platforms for gene addition

In addition to γRV and LV platforms, other integrating gene transfer systems under investigation include transposons. Trials using sleeping beauty and piggyBac systems (PB) have been underway for CAR T cell therapies [84], and rely on co-delivery of transposases and CAR gene expression cassettes flanked by appropriate recombination sequences. New-onset T cell lymphomas were reported in two patients receiving engineered T cells using *PiggyBac* transposons for expression of CAR19 [85]. Extensive investigations were carried out to uncover possible mechanistic origins of these T cell malignancies [86]. Despite elevated transposon copy numbers observed in both normal CAR T cells and transformed cells, these events did not appear directly linked to integration sites and there was no evidence that clonal expansion was driven by a CAR-mediated activation. Next-generation sequencing detected several copy number variations in proto-oncogene and tumour-suppressor genes and transcriptome analysis identified associated clusters of upregulated and downregulated genes, but no obvious links to PB integration sites. The high number of integrated transposons in these two cases suggested a link between transposition and genotoxicity, but a complete understanding of underlying pathways is still lacking [87]. However, the experience aptly reveals that mature T cells can be prone to malignant transformation under particular circumstances and this is of importance when considering how and when emerging genome editing platforms are applied.

### Genome editing tools and risk of genetic aberrations

Platforms including Zinc Finger Nuclease (ZFNS) [88], transcription activator-like effector nucleases (TALENs) [89], Homing endonucleases [90], clustered regularly interspaced short palindromic repeats (CRISPR-Cas9) [91] and base editing [92] have been deployed for adoptive immunotherapy [93–96]. Areas for consideration around genotoxicity risks include unwanted gene disruption, chromosomal loss, translocations or other karyotype changes and can be framed as ‘on-target’ events at sites of expected activity, or ‘off-target’ and possibly promiscuous effects with unexpected DNA changes [97, 98]. Previously, TALEN editing for generation of “universal” CAR-T cells (disrupted for TCR and CD52), was associated with around 4–6% residual translocations at the end of manipulation [99]. A recent report documented persistence of chromosomal translocations in a patient treated with TALEN-edited “universal” CAR-T cells [100], who had developed bone marrow aplasia after treatment with allogenic CAR-19 cells and had emergence of a clonal T cells expansion harbouring an inversion of chromosome 14. The abnormality was attributed to spontaneous V(D)J recombination, involving incidental recombination-activating genes (RAG) recombination sequences rather than TALEN mediated effects. Similar frequencies of chromosomal changes have been documented after CRISPR-Cas9 editing in autologous recombinant TCR studies, and in-depth informatics detected frequent chromosomal changes including aneuploidy. A relatively high frequency of karyotype aberrations was reported after lentiviral transfer of TCR to autologous T cells edited at T Cell Receptor Alpha Constant (TRAC) and Programmed Death 1 (PD1) genes using CRISPR/Cas9. Single-cell RNA sequencing showed a high frequent aneuploidy and chromosomes cleavage at the TRAC and PD1 target sites [98]. Recently, there have been suggestions that the manner of T cell manipulation, including of the timing of T cell activation and CRISPR-Cas9 editing, can be modified to reduce the frequency of such events [6, 98, 101].

Homo or heterozygous deletion of Programmed Cell Death 1 (PDCD1) gene, encoding for PD-1, a major regulator of T-cell exhaustion, promote aberrant T-cell proliferation in animal models [102]. Despite the potential role of PD-1 as a haploinsufficient suppressor of T-cell lymphomagenesis, no T cell malignant transformation have been reported in clinical studies targeting PD-1 to date. Other examples where potential concerns may arise include CRISPR work editing TET2 for possible epigenetic programming to enhance CAR T cell function. After editing, CAR T cells with biallelic TET2 disruption displayed clonal expansions, much like those reported after LV mediated disruption [55], with tissue infiltration in animal models and effects attributed to sustained expression of the AP-1 factor BATF3 and MYC-dependent proliferation [103, 104]. Manipulation of other epigenetic pathways, for example through deletion of DNMT3A in T cells is of interest for increased functionality and exhaustion resistance in other models [105], but somatic mutations in DNMT3A have been reported in T cell derived malignancy [71, 73, 74], as discussed above.

Base Editing (BE) and prime editing (PE) build on canonical CRISPR-Cas technology to deliver single nucleotide conversions or insertion of short reverse transcribed sequences and are reaching the testing in clinic [93, 94]. While multiplex editing in T cells triggered easily detectable translocations, base editors were shown to reduce risks related to DNA cleavage, with virtually absent translocations [92]. Engineered adenine deaminase enzymes for A > G conversions and evolved versions of cytidine deaminase for C > T editing are amongst variants that hold promise for forthcoming developments, with low levels of off-target activity and residual DNA breakage. Laboratory studies using human CD34+ and T cells have compared BE and PE editing strategies and quantified on- and off- target effects, activation of p53, innate sensing editing efficiency, transcriptional and genome-wide genotoxicity during hematopoietic repopulation in humanised mice [106]. Currently there is a paucity of direct in human data, although in non-human primate there have been suggestions that gene editing followed by homology direct repair (HDR) system might result in reduced engraftment of manipulated HSC, when compared with canonical LV transduced HSC, with also a marked skewing towards oligoclonality. The issue of long-term dysfunction of genome edited HSC has been under consideration in human trials using AAV mediated HDR delivery of a repair template to correct HSC in SCD [107]. Prolonged cytopenia and lack of engraftment of manipulated cells occurred in the first patient enroled [108], an issue that was not encountered in studies using CRISPR mediated NHEJ for disruption of the enhancer region of BCL11A to induce re-expression of γ-globin and production of foetal haemoglobin. In 2023 a licenced version of the product, Exagamglogene autotemcel (Casgevy®) became the first CRISPR-based therapy to be approved for treatment of beta-thalassemia and sickle cell disease [109, 110], and long term tracking will be essential to map and define the safety profile of the approach.

## Conclusions and outlook

Increased awareness around mechanisms of genotoxicity have improved safety vector platforms as wider applications of genetically modified blood stem cells and immune cell therapies have continued to emerge. Current guidelines recommend long-term monitoring of patients receiving advanced medicinal products and after gene therapy follow-up of 15 years to monitor for potential genotoxicity effects is widely underway. Complications uncovered during early clinical trials using γRV have been largely mitigated by switching to SIN-LV for gene transfer to HSC, and there are emerging clinical indicators that certain vector features, including the incorporation of insulators and choice of internal promoter need careful consideration. The inclusion of LTR derived promotor /enhancers, often chosen for their ability to support high levels of transgene expression, may be particularly hazardous. Nor should differentiated cells such as T cells be considered impervious to transformation risk- as larger numbers of subjects are dosed, low frequency events may be uncovered and understanding contributions from vector systems, transgenes and underlying predispositions will be critical.

Experience so far has set the scene for a wide range of further applications and exploitations of gene engineered cells therapies and emphasised the importance of careful monitoring and sample archiving over the long term.
